# Deactivation of Signal Transducer and Activator of Transcription 3 Reverses Chemotherapeutics Resistance of Leukemia Cells via Down-Regulating P-gp

**DOI:** 10.1371/journal.pone.0020965

**Published:** 2011-06-06

**Authors:** Xulong Zhang, Weihua Xiao, Lihua Wang, Zhigang Tian, Jian Zhang

**Affiliations:** 1 School of Pharmaceutical Sciences, Shandong University, Jinan, China; 2 Department of Immunology, School of Basic Medical Sciences, Capital Medical University, Beijing, China; 3 Hefei National Laboratory for Physical Sciences at Microscale and School of Life Sciences, University of Science and Technology of China, Hefei, China; Texas A&M Universityaddress

## Abstract

Multidrug resistance (MDR) caused by overexpression of p-glycoprotein is a major obstacle in chemotherapy of malignant cancer, which usually is characterized by constitutive activation of signal transducer and activator of transcription 3 (STAT3), but their relation between MDR and STAT3 remains unclear. Here, we showed that STAT3 was overexpressed and highly activated in adriamycin-resistant K562/A02 cells compared with its parental K562 cells. Blockade of activation of STAT3 by STAT3 decoy oligodeoxynucleotide (ODN) promoted the accumulation and increased their sensitivity to adriamycin by down-regulating transcription of *mdr1* and expression of P-gp, which were further confirmed by using STAT3-specific inhibitor JSI-124. Inhibition of STAT3 could also decrease *mdr1* promoter mediated luciferase expression by using *mdr1* promoter luciferase reporter construct. Otherwise, activation of STAT3 by STAT3C improved *mdr1* transcription and P-gp expression. The ChIP results demonstrated that STAT3 could bind to the potential promoter region of *mdr1*, and STAT3 decoy depressed the binding. Further mutation assay show +64∼+72 region could be the STAT3 binding site. Our data demonstrate a role of STAT3 in regulation of *mdr1* gene expression in myeloid leukemia and suggest that STAT3 may be a promising therapeutic target for overcoming MDR resistance in myeloid leukemia.

## Introduction

Leukemia is the sixth most lethal cancer accounting for 4% of all cancers, and the most common childhood cancer accounting for 32.6% of all childhood cancers. Chemotherapy is the most effective method for leukemia treatment. However, a 5-year relative survival ratio is only about 50% due to MDR (American Cancer Society). Several potential molecular or cellular mechanisms responsible for MDR have been elucidated. The over-expression of P-gp, encoded by the ATP Binding Cassette B1 (ABCB1, also known as *mdr1*) gene, is considered as one of the major obstacles to successful cancer chemotherapy.[1] P-gp is a trans-membrane glycoprotein which reduces intracellular drug concentrations by pumping drugs out of the cells.[2] Numerous studies have confirmed that the expression of P-gp is an adverse prognostic factor for complete remission and survival in adult AML.[3], [4] Unfortunately, the efflux-pump modulators used in clinic have been proved disappointing. This is at least to a certain extent because the fundamental molecular mechanisms driving MDR phenotype of leukemia cells remain obscure. Therefore, the characterization of signaling pathways about MDR leukemia is very important for designing rational novel therapies.

STAT3 is constitutively activated in diverse human cancers including primary leukemia and leukemia derived cell lines.[5], [6] The activity of STAT3 has been implicated in tumor cell survival, inhibition of apoptosis, proliferation, motility, angiogenesis, invasion and metastasis.[6] The constantly activated STAT3 contributes to oncogenesis by up-regulation of genes encoding bcl-xl, cyclin D1, mcl-1, and VEGF, IL-10, TGF-β et al.[6] Our previous study also showed that blockage of STAT3 by decoy ODN decreased proliferation and induced apoptosis by down-regulating its target genes *in vitro* and *in vivo.* [7], [8]

Constitutive activation of STAT3 has been shown to confer resistance to chemotherapy-induced apoptosis in some malignancies. For example, inhibition of Src not only sensitized ovarian cancer cells to chemotherapeutic agents but also re-sensitized paclitaxel-resistant cells to paclitaxel.[9] Blockade of STAT3 with the STAT3 decoy ODN augmented apoptosis when combined with cisplatin both *in vitro* and *in vivo.* [10] Inhibition of STAT3 activity enhanced chemosensitivity in hepatocellular, stomach carcinoma and melanoma.[11], [12], [13] Additionally, some researchers demonstrated that multidrug resistance was consistent with *STAT3* mRNA over-expression in cisplatin-resistant lung cancer cells [14] and STAT3 activity specifically elevated in drug-resistant neuroblastoma and ovarian cells, while not in relevant drug-sensitive cells.[15] Recently, it was demonstrated that inhibition of STAT3 effectively enhanced multidrug sensitivity *via* inhibiting Nanog/STAT3-mediated *mdr1* gene expression in both MCF-7 cells and SK-OV-3.ipl cells.[16] In clinic, STAT3 is highly activated in drug non-sensitive advanced tumors. All these findings suggested that STAT3 might be associated with MDR and their relation needed to be defined, and this study therefore aimed at deciphering the potential role of STAT3 in chemotherapy resistance in leukemia.

In this study, the results presented that elevated expression and constitutive activation of STAT3 was observed in adriamycin-resistant K562/A02 cells. Inhibition of STAT3 increased the sensitivity of K562/A02 cells to adriamycin by down-regulating *mdr1*. All these findings suggested that STAT3 signaling may be an interesting target for reverting (or preventing) chemoresistance in myeloid leukemia.

## Materials and Methods

### Cell culture

The human leukemia sensitive parental K562 cell was obtained from the American Type Culture Collection (Rockville, MD) and adriamycin-selected MDR K562/A02 subline was obtained from the Institute of Hematology of Chinese Academy of Medical Sciences (Tianjin, China).[17] K562/A02 displayed 310-flod resistance to adriamycin.[18] MCF-7 and MCF-7/ADR was obtained from Cancer Institute & Hospital of Chinese Academy of Medical Sciences. Cells were cultured in DEME medium (GIBCO/BRL) containing 10% (V/V) fetal calf serum (FCS, GIBCO/BRL), 100IU/ml penicillin and 100 µg/ml streptomycin in 5% CO_2_ at 37°C. K562/A02 and MCF-7/ADR cells were routinely maintained in medium containing 200 µg/L or 1mg/L adriamycin, respectively.[17] We concluded that the comparison of K562 and K562/A02 constitutes a valid model to study induced resistance for classical chemotherapy.

### Antibodies and reagents

Anti-STAT3, anti-phospho-specific STAT3 (Tyr705, Ser727), anti-β-actin antibodies and horseradish peroxidase-conjugated second antibody were purchased from Cell Signaling Technology. Anti-P-gp was purchased from Santa Cruz (Santa Cruz Biotechnology, Inc.). STAT3 inhibitor JSI-124 was purchased from Calbiochem (Darmstadt, Germany). STAT3 decoy and scrambled ODN were synthesized and purified as previously.[7], [8] The STAT3 decoy ODN sequence was 5′-CATTTCCCGTAAATC-3′, 3′-GTAAAGGGCATTTAG-5′ and the scrambled ODN sequence was 5′-CATCTTGCCAATATC-3′, 3′-GTAGAACGGTTATAG-5′. The sense and antisense strands were annealed and purified by HPLC.

### RNA isolation and real-time PCR assay

RT-PCR was performed as manufacturer's instructions. Briefly, total RNA was isolated using TRIzol (Invitrogen, Carlsbad, CA) as previous described.[7] And cDNA were synthesis using the M-MLV reverse transcriptase (Invitrogen, Carlsbad, CA).[7] Quantitative real-time PCR was performed by using SYBR Green kit (Bio-rad) on Opticon Monitor (Bio-rad). The assays were initiated with 15 min at 95°C, and then 40 cycle of 15 s at 94°C, 1 min at 58°C, 1 min at 72°C. The primers sequences were listed in supplementary table S1. Relative mRNA expression levels of interested gene were calculated using the 2^-ΔΔCt^ method.

### *Mdr1* promoter luciferase reporter plasmid construction

The pGL3-mdr1 promoter luciferase reporter plasmid contains a 617 bp fragment of *mdr1* genomic sequence upstream of the initiating ATG. It includes potential STAT3 binding sites as verified by ChIP. This fragment was amplified using the following primers: 5′- CTAGGTACCTCAAAGGTGTTAGGAAGCAGAAAG-3′ and 5′-CAGGCTAGCTGAAAGCCTGACACTTGGGAAC-3′, and subcloned into KpnI and NheI sites of pGL3-Basic vector (Promega, Madison, WI). The reporter plasmids were controlled by sequencing. For the mutation of potential STAT3 binding site, overlap-PCR was used and the mutated sequence was shown in table 1.

**Table 1:** Mutation sequence of potential STAT3 binding sites.

	Potential binding site 1 (-330∼-322)	Potential binding site 2 (+64∼+72)
Wide type	AAT**TTCCCTTAA**CTA	TCT**TTCCACTAA**AGT
STAT3M1	AAT**CGCCCTTGA**CTA	
STAT3M2		TCT**CGCCACTGA**AGT
Consensus sequence	**5’-TTMXXXDMA-3’,** where D is A, G, or T and M is A or C.	

In bold is the consensus SIE motif, and the bold underlined letters correspond to the mutated bases from the SIE motif.

### Dual-luciferase reporter plasmid assay

6.25 ng pGL3-STAT3-TK-luciferase reporter vector or pGL3-TK-luciferase control construct with 0.625 ng of pRL-TK were co-transfected by lipofectamine 2000 (Invitrogen, Carlsbad, CA). After 5 h, cells were cultured in fresh medium for 24 h. The activities of luciferases are measured by Dual-Glo^TM^ Luciferase Assay System (Promega, Madison, WI) according to the manufacturer's instruction by a luminometer (PerkinElmer 1420). To correct for variations in transfection efficiencies, the firefly luciferase values were normalized against renilla luciferases. For STAT3 decoy ODN inhibition assay, the plasmids and decoy/scrambled ODN (200 nM) were co-transfected. Triplicates were performed for each transfection study and three individual experiments were conducted.

### Drug accumulation

Accumulations of adriamycin in drug sensitive or resistant cells were measured by using flow cytometry. 3×10^5^ cells in 1 ml were plated in 6-well plate. After 24 h, cells were pretreated with indicated concentrations of JSI-124 or transfected with 200 nM decoy/scrambled ODN for 24 h, and then exposed with adriamycin or rhodamine 123 for 90 min at 37°C. After three washes with ice-cold PBS, the cells were resuspended and detected with flow cytometer. The ratio of MFI over control was calculated as follow: [MFI of adriamycin (Rh123) in treatment group]/[MFI of adriamycin (Rh123) in control group].

### Cell proliferation assay

Cell proliferation was examined by tritiated thymidine (^3^H-dThd) incorporation or CCK-8 kit. The K562/A02 and K562 cells (4×10^4^/well) were cultured in 24-well plate for 24 h, and JSI-124 was added into the wells at the indicated concentrations and incubated for another 18 h or 42 h, and then pulsed for the remaining 6 h with [^3^H]thymidine (3.75 µCi/1 ml) (Amersha Biosciences, UK). [^3^H]Thymidine incorporation was analyzed by liquid scintillation counting.

For CCK-8 assay, 1×10^4^ cells/well in 100-µl medium were cultured in 96-well plate for 24 h. The K562/A02 and K562 cells were treated with different concentrations of adriamycin (or daunorubicin) combined with 1 µM JSI-124. Additionally, the cells were also transfected with 200 nM STAT3 decoy/scrambled ODN by lipofectamine 2000 for 6 h, and then refreshed with medium containing indicated concentration of adriamycin. After cultured for 48 h, 10 µl of Cell Counting Kit-8 reagent (Dojindo Molecular Technologies, Gaithersburg, MD) was added to each well and incubated for another 3 h at 37°C. The plates were read at A_450 nm-630 nm_ using a microplate reader (Bio-Rad). The inhibition ratio was calculated by following formula: inhibition (%)  =  (1-experimental OD/control OD) × 100%. An average of at least three triplicates from three separate experiments was calculated. The relative inhibition rates were represented with the O.D. value of the control group set as zero.

### Western blots

As described previously, [7] the extracts were resolved by SDS-PAGE and transferred onto PVDF membranes (Millipore, Bedford, MA). The bands were detected with the correspondence antibodies.

### Chromatin immunoprecipitation (ChIP) assays

K562/A02 cells were transfected with 200 nM STAT3 decoy/scrambled ODN for 12 h. ChIP was done using a ChIP assay kit (Upstate Biotechnology, Temecula, CA) according to the manufacturer's instructions. Briefly, proteins were cross-linked with DNA, and sheared using a sonicator. STAT3/DNA complexes were precipitated either by anti-STAT3 antibody or by rabbit IgG as the negative control. And then the anti-STAT3/STAT3/DNA complex was collected by Protein G Agarose. The amount of immunoprecipitated DNA was assessed by semiquantitative PCR using primers spanning the different potential STAT3 binding region in *mdr1* promoter as shown in table 2. The conditions for PCR reactions were as follows: 94°C for 5 min, 35 cycles at 94°C for 30 s, 57°C for 40 s, 72°C for 40 s, and a final extension at 72°C for 5 min. Enrichment in each immunoprecipitation was determined by quantifying the intensities of the PCR product in immunoprecipitated DNA versus 10% input DNA.

**Table 2:** The primer sequences for ChIP.

		Primer sequence
ChIP-1	sense	5’-CAGCAATGAATACCTTTGGCTG-3’
	antisense	5’-CTAATAGAACCGAGCAAAAGATGC-3’
ChIP-2	sense	5’-GTTCAAGATCAGCCTAGTCAATGTG-3’
	antisense	5’-TACCACTCCGCAATCCTACTTTTC-3’
ChIP-3	sense	5’-CTCTTTGGACTGAGTGGAAACTTG-3’
	antisense	5’-TTGCCCTCCTTAGCATTCTTTG-3’
ChIP-4	sense	5’-GGTTAGCACCTTACCTGCTGTG-3’
	antisense	5’-AATCTGCACCTTCTTGTCCTCC-3’
ChIP-5	sense	5’-CTTTTCAAAGGTGTTAGGAAGCAG-3’
	antisense	5’-GAATTTCCAGGAGGAATGTTCTG-3’
ChIP-6	sense	5’-TCAACCTGTTTCGCAGTTTCTC-3’
	antisense	5’-TAGTAGCTCCCAGCTTTGCGTG-3’

### Statistical analysis

All of the values are presented as the mean ± SD for three or more individual experiments. SPSS software (version 11.5, SPSS Inc.) was used to test for significance. Statistical significance was determined as *P<0.01 and ^#^ *P*<0.05 compared with control. NS  =  non-significant.

## Results

### STAT3 was over-activated in adriamycin-resistant K562/A02 cells

**Figure 1:**
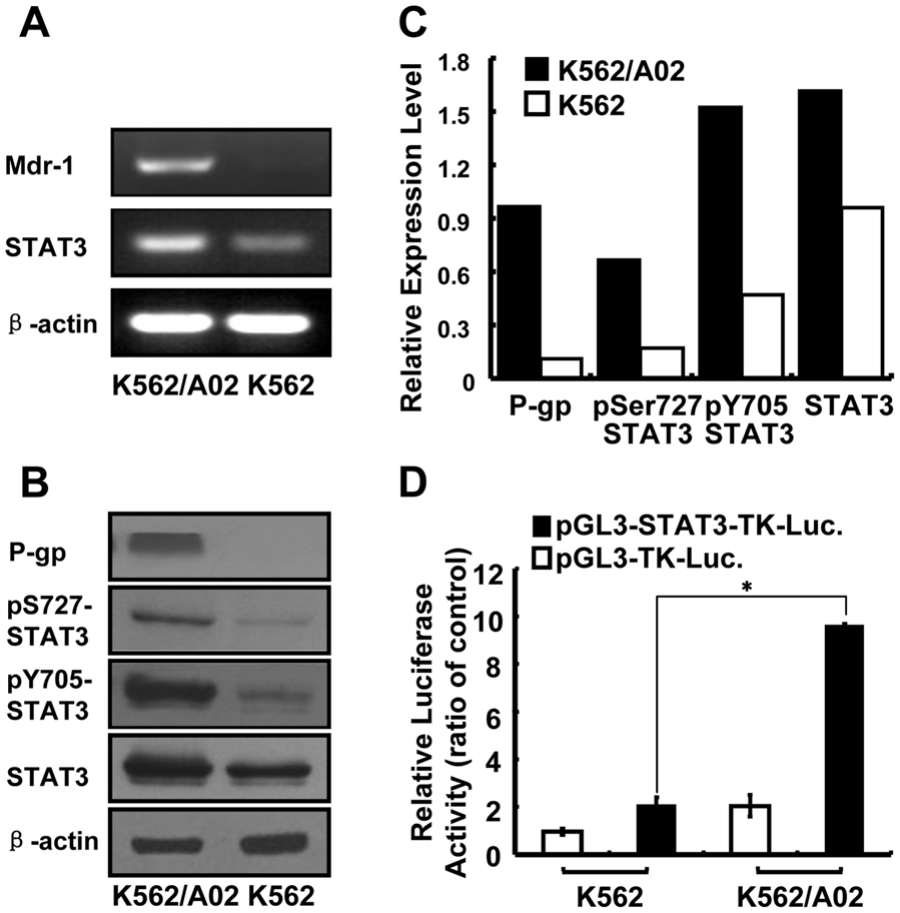
STAT3 activation in adriamycin-resistant K562/A02 cells. (**A**) RT-PCR analysis of STAT3 and *mdr1* in K562/A02 cells and K562 cells. (**B**) Western blot analysis of total STAT3, phosphorylation of STAT3 and P-gp. (**C**) The histogram presented the relative expression levels of each protein after normalization to their corresponding internal control. (**D**) STAT3 activation was detected by using luciferase reporter construct assay. Both cells were co-transfected with pGL3-STAT3-TK-Luciferase or pGL3-TK-Luciferase and pRL-TK-luciferase, and then luciferase activity was measured.

The activity of STAT3 is usually higher in MDR malignancies, and inhibition of STAT3 activity might reverse the chemo-resistance.[15] In this study, we concluded that the comparison of K562 and K562/A02 constitutes a valid model to study induced resistance for classical chemotherapy.[17], [18] It was observed that mRNA level of STAT3 was higher in adriamycin-resistant K562/A02 cells (Fig. 1A), total STAT3 protein expression was increased, and the phosphorylation levels of STAT3 (Y705, S727) were also much higher in K562/A02 cells (Fig. 1B, C). By using STAT3-dependent luciferase reporter construct, we observed that higher STAT3 activity was shown in K562/A02 (Fig. 1D), which was consistent with the results of western blot. P-gp was also strongly expressed in drug-resistant cells but not in drug-sensitive cells at mRNA levels (Fig. 1A) and protein levels (Fig. 1B, C). Meanwhile, STAT3 target gene, such as *bcl-xl*, was also up-regulated in K562/A02 cells (data not shown). To further confirm the results, adriamycin-resistant MCF-7/ADR and sensitive MCF-7 cancer cell line were analyzed. Compared with MCF-7 cells, the expression and phosphorylated levels of STAT3 were higher in adriamycin**-**resistant MCF-7/ADR cells (Fig. S1A, B), which was concomitant with strongly expressed P-gp (Fig. S1A, B). Overall, these data show that the MDR phenotype is accompanied by constitutively activated STAT3 signaling pathway.

### STAT3 decoy ODN reversed adriamycin resistance through downregulation of *mdr1* in K562/A02 cells

**Figure 2:**
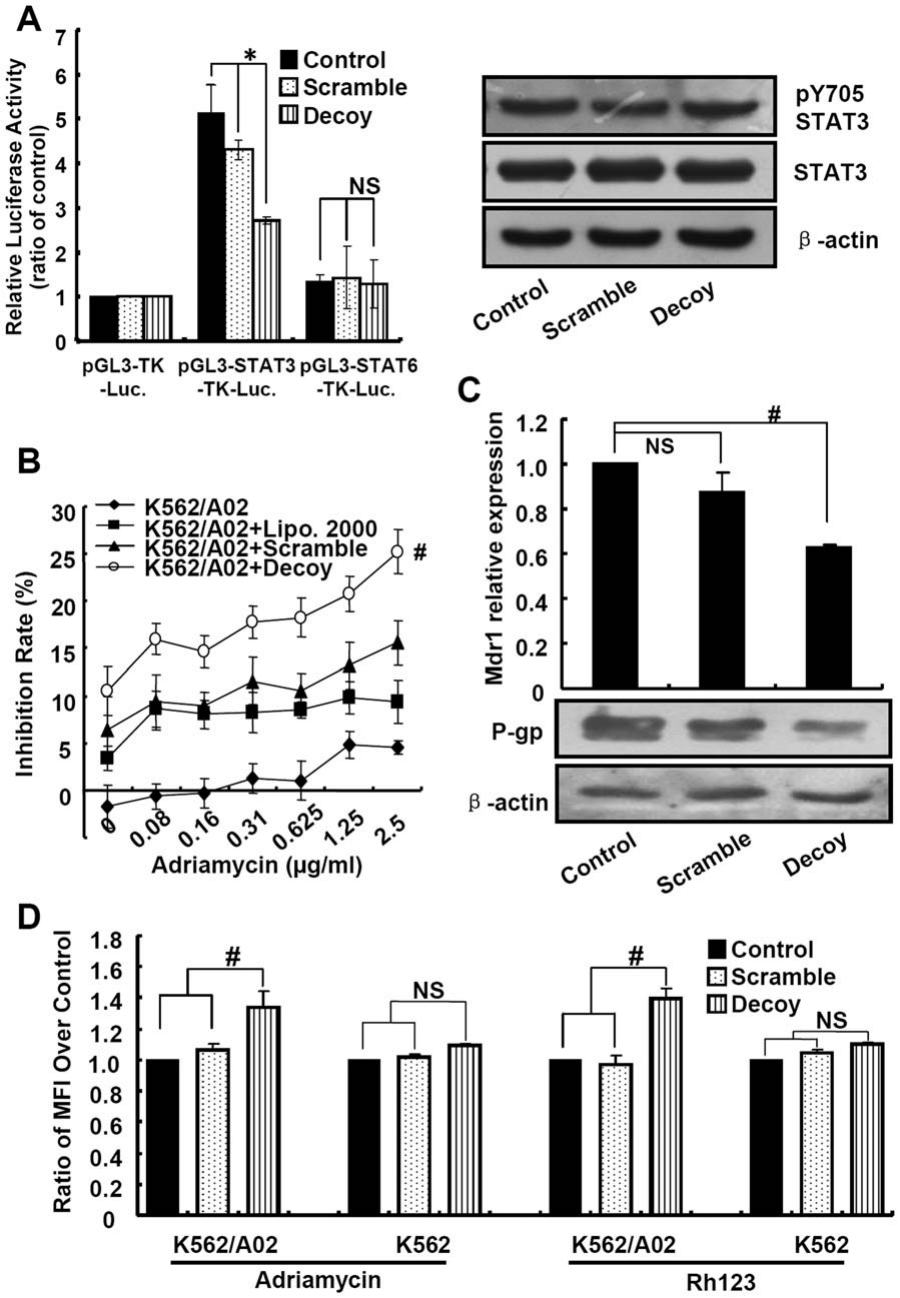
STAT3 decoy ODN increased the drug sensitivity *via* inhibiting the transcription of *mdr1* and increasing the accumulation of adriamycin. (**A**) Luciferase reporter assay and western blot were used to detect the STAT3 activation and expression after inhibiting by decoy ODN. K562/A02 cells were co-transfected with STAT3 or STAT6-Luciferase reporter construct and decoy/scrambled ODN, and then luciferase activity was measured. Total STAT3, pY705 STAT3 were detected by western blotting. (**B**) STAT3 decoy ODN increased the sensitivity of K562/A02 cells to adriamycin. K562/A02 cells were transfected with 200nM STAT3 decoy or scrambled ODN. After 6h, different concentrations of adriamycin were added and incubated for 48h followed by the CCK-8 assay. (**C**) STAT3 decoy ODN inhibited the transcription and expression of *mdr1*. K562/A02 cells were transfected with STAT3 decoy or scrambled ODN, and mRNA and protein levels of *mdr1* were measured by real time-PCR and western blotting. (**D**) STAT3 decoy ODN increased the accumulation of adriamycin and rhodamine 123 into K562/A02 cells. K562/A02 and K562 cells were pretreated with the STAT3 decoy or scrambled ODN, and then treated with adriamycin or rhodamine 123. Fluorescence intensity of adriamycin and rhodamine 123 were determined by flow cytometry.

STAT3 decoy ODN was used as an efficient method to inhibit the activity of STAT3.[7], [8] To assess whether STAT3 decoy ODN blocks the transcription activity of STAT3 in K562/A02 cells, the STAT3 activity was analyzed by dual-luciferase reporter assay after co-transfection of reporter plasmid and decoy/scrambled ODN. As shown in Fig. 2A, STAT3 decoy ODN reduced transcriptional activity of STAT3 compared with scrambled ODN, while it could not block the activation of STAT6, indicating the specificity of STAT3 decoy ODN (Fig. 2A). However, the expression of STAT3 and the phosphorylation of Y705-STAT3 showed no detectable changes after ODN treatment, which were consistent with previous report (Fig. 2A).[19]

To elucidate the relationship between STAT3 and MDR, we firstly investigated whether blocking the activation of STAT3 could reverse adriamycin resistance under our experimental conditions. As shown in Fig. 2B, blockade of STAT3 activation with STAT3 decoy ODN significantly increased the inhibition rate of K562/A02 cells by adriamycin, indicated that the cytotoxicity of adriamycin was promoted through inhibition of STAT3. Meanwhile, the similar results were obtained in MCF-7/ADR cells (Fig. S2A). Secondly, we analyzed the transcription and expression levels of *mdr1*. As shown in Fig. 2C, STAT3 decoy ODN but not scrambled ODN obviously decreased the transcription of *mdr1* in real-time PCR analysis. Compared with control, *mdr1* transcripts were decreased to 62.7% by treating with 200 nM STAT3 decoy ODN (*p*<0.05), whereas other MDR-associated genes, such as MRP1 and BCRP1, showed no obvious changes (data not shown). Simultaneously, the expression of P-gp was also reduced to 42.7% when compared with control (Fig. 2C). Similar phenomena were observed in MCF-7/ADR cells (Fig. S2B). Finally, the intracellular adriamycin accumulation was explored after STAT3 decoy ODN treatment by using flow cytometry method. The results demonstrated that the accumulation of adriamycin was enhanced in K562/A02 or MCF-7/ADR cells treated with STAT3 decoy ODN, but did not in K562 or MCF-7 cells (Fig. 2D, Fig. S2C). Rhodamine-123, P-gp substrate, was also used to evaluate intracellular accumulation, and the MFI of rhodamine-123 in cells treated with STAT3 decoy ODN was elevated (Fig. 2D), revealing that P-gp-medicated rhodamine-123 efflux can be inhibited by blocking of STAT3 activation. These results suggested that deactivation of STAT3 could increase drug accumulation by suppressing *mdr1* transcription.

### Inhibiting STAT3 led to down-regulation of *mdr1* and increased K562/A02 drug sensitivity

**Figure 3:**
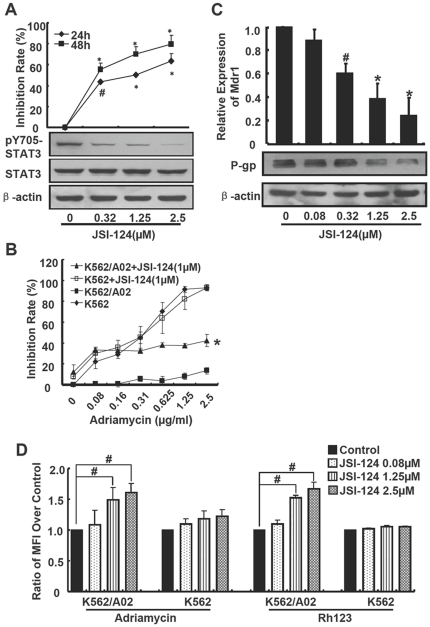
JSI-124 increased the sensitivity of K562/A02 cells to adriamycin through down-regulation of *mdr1.* (**A**) JSI-124 inhibited phosphorylation of STAT3 and the proliferation of K562/A02 cells. Cells were cultured in medium containing 1 µg/ml adriamycin and different concentrations of JSI-124, and proliferation was detected with [^3^H] thymidine incorporation. STAT3 and pY705STAT3 were measured by western blotting. (**B**) The cells were cultured with different concentrations of adriamycin combined with 1 µM of JSI-124 followed by the CCK-8 assay. (**C**) JSI-124 inhibited the transcription of *mdr1* and expression of P-gp. After being treated with JSI-124 as indicated concentrations for 24 h, mRNA level of *mdr1* in K562/A02 cells was detected using real time-PCR. P-gp was examined by western blotting after being treated for 36h. (**D**) JSI-124 increased the accumulation of adriamycin into K562/A02 cells. Cells were pretreated with JSI-124 for 24h, and then treated with adriamycin or rhodamine 123 for 90min. Fluorescence intensity were determined by flow cytometry.

To further confirm our observation that deactivation of STAT3 could reverse the drug resistance, JSI-124, a selective inhibitor for JAK/STAT3, was further used.[20] JSI-124 down-regulated the phosphorylation of STAT3 in K562/A02 cells in a dose-dependent manner but the total STAT3 level was not influenced (Fig. 3A), and JSI-124 dose- and time-dependently inhibited proliferation of K562/A02 cells when co-treated with 1 µg/ml adriamycin (Fig. 3A). After treated for 24 h, the inhibition ratios were 43%, 50% and 63% for 0.32 µM, 1.25 µM and 2.5 µM JSI-124, respectively. And the inhibition ratios rose to 55%, 70% and 79% after treated for 48 h. When treated with different concentrations of adriamycin combined with 1 µM JSI-124 for 48 h, JSI-124 induced about 8-fold reduction of IC_50_ in adriamycin-resistant K562/A02 cells, but did not in K562 cells (Fig. 3B). Similarly, the inhibitory effect of adriamycin on MCF-7/ADR cell proliferation was augmented by JSI-124 (Fig. S3A).

At the same time, *mdr1* transcripts were decreased by 62% and 75% when treated with 1.25 µM and 2.5 µM JSI-124 respectively (*p*<0.01) (Fig. 3C), and the expression level of P-gp was also decreased by JSI-124 in a dose-dependent manner. P-gp was reduced by 53% and 71% after treated with 1.25 µM and 2.5 µM JSI-124 for 36 h, respectively (Fig. 3C). Compared with control, JSI-124 dose-dependently improved the accumulation of adriamycin or rhodamine-123 in K562/A02 cells, but not in K562 cells (Fig. 3D). Consistent with these above, JSI-124 also dose-dependently attenuated the expression of mdr-1 and P-gp in MCF-7/ADR cells (*p*<0.05) (Fig. S3B), and enhanced the accumulation of adriamycin in MCF-7/ADR cells, but not in MCF-7 cells (Fig. S3C).

Since K562/A02 cells were also strongly resistant to daunorubicin, the effect of JSI-124 on daunorubicin treated K562/A02 cells was also investigated. We found that JSI-124 significantly increased the sensitivity of K562/A02 cells to daunorubicin, and induced about 62-fold reduction of IC_50_ in K562/A02 cells (Fig S4). Thus, our data shows that drug sensitivity could be increased by suppression of STAT3 activation in multi-drug resistance cells.

### *Mdr1* was transcriptional regulated by STAT3

**Figure 4:**
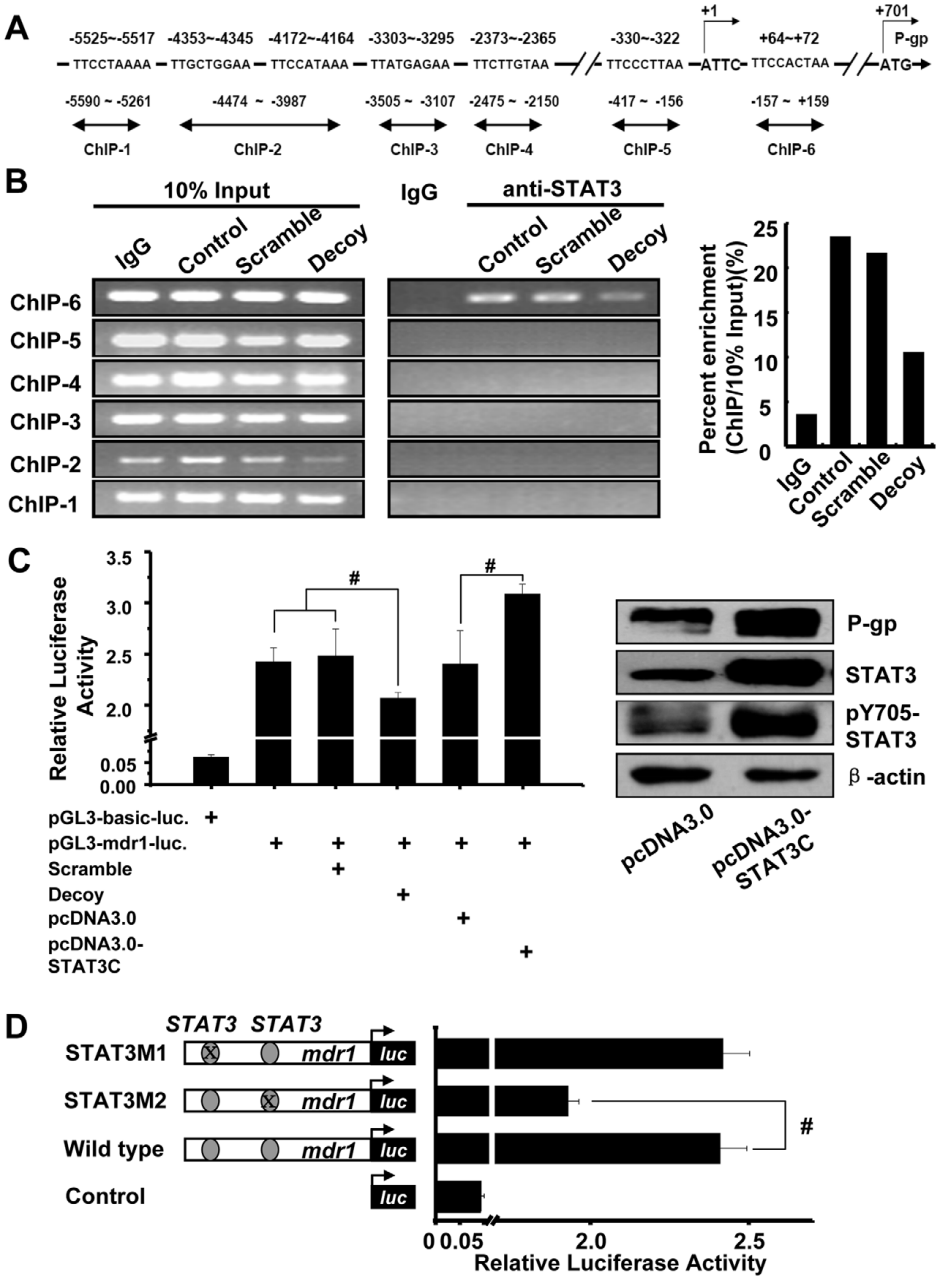
*Mdr1* was transcriptional regulated by STAT3. (**A**) The sketch map of predicted STAT3 binding sites within *mdr1* promoter. The ATTC, transcriptional start site was as +1, and ATG is as translation start site. The putative STAT3 binding sites are shown and their locations are labeled. Six sets of PCR primers, designated as ChIP-1, ChIP-2, ChIP-3, ChIP-4, ChIP-5 and ChIP-6, were used for amplification of the potential STAT3 binding sites by ChIP assay, respectively. (**B**) ChIP assay was performed using anti-STAT3 antibody or irrelevant IgG antibody as negative control. Input: total genomic DNA used as control for the PCR. The histogram represented the intensities of the PCR product in immunoprecipitated DNA versus 10% input DNA of ChIP-6 primer. (**C**) Luciferase reporter assay was used to detect the STAT3-dependent transcriptional activation. pGL3-*mdr1* promoter luciferase construct alone or with STAT3 decoy/scrambled ODN or with pcDNA3.0, STAT3C plasmid were co-transfected and luciferase activity was detected. STAT3C increased the total and activated STAT3, and promoted the expression of P-gp. K562/A02 cells were transfected with STAT3C for 40 h. Proteins were extracted and examined by western blotting. (**D**) Identification of STAT3 binding site in *mdr1* promoter. Constitution of pGL3-*mdr1*-luciferase reporter and the mutated plasmid were described as in Materials and Methods, and their luciferase activity was assayed.

The possibility that STAT3 participated in the regulation of *mdr1* transcription was explored. Computer-assisted searches of potential STAT3 binding sites within *mdr1* promoter region revealed that there were seven possible sites containing putative STAT3 DNA binding elements by using TFSEARCH and TRANSFAC database (predicting transcription factor binding sites), these regions are located at different position of *mdr1* promoter (Fig. 4A). As STAT3 binding site, the consensus sequence 5′-TTMXXXDMA-3′ was used for comparison.[21], [22]

To further identify whether STAT3 could bind to *mdr1* promoter in K562/A02 cells, ChIP assays were performed, immunoprecipitated and inputted DNA were amplified using the primers covering the suggested STAT3 binding sites as shown in Fig. 4A. Firstly, the ChIP results exhibited that STAT3 could bind to the consensus sequence located at -157∼+159 of *mdr1* promoter, and STAT3 decoy ODN depressed the binding (Fig. 4B). However, no binding of STAT3 was found in other regions of *mdr1* promoter. Next, pGL3-*mdr1*-promoter luciferase reporter vector which contains the two potential STAT3 binding sites was constructed, and reporter assay was used to confirm whether STAT3 participated in the transcription of *mdr1*. As shown in Fig. 4C, *mdr1* promoter mediated luciferase expression was effectively initiated, STAT3 decoy ODN but not scrambled ODN decreased the luciferase activity. Meanwhile, co-transfection with STAT3C plasmid, which expressed constitutively activated STAT3 (Fig. 4C right), obviously increased *mdr1* promoter mediated luciferase activity in K562/A02 cells, accompanied by the enhanced expression of P-gp (Fig. 4C right). Finally, to more definitively map the STAT3 binding site and its functionally relevant in *mdr1* promoter, the potential STAT3 binding region at −330∼−322 and +64∼+72 were mutated separately, and their luciferase activity were assayed. The results showed that mutation at +64∼+72 region, but not −330∼−322 region, lead obviously reduction of the transcription activity (Fig. 4D). These findings indicated that the DNA sequence at +64∼+72 region was recognized by STAT3 and responsible for *mdr1* transcription. All these results suggested that STAT3 could bind with *mdr1* promoter sequence and participated in the regulation of *mdr1* transcription.

## Discussion

The effectiveness of chemotherapy is seriously limited by MDR which mediated mainly by P-gp. Since the early 1980s, some compounds were found to overcome P-gp-mediated MDR. However, they had only limited success in clinical trials. Therefore, the characterization of signaling pathways sustaining MDR is thus essential for designing rational novel therapies for MDR leukemia. This notion is supported by the data in the present study showing that inhibition of STAT3 pathway down-regulated P-gp and partly reversed P-gp mediated MDR in leukemia cells.

Our previous cumulative evidences supported that activated STAT3 might be a target for anti-tumor strategy and STAT3 decoy ODN could be an ideal tool for inhibiting STAT3.[7], [8], [23] In this study, it was demonstrated that STAT3 was more activated in adriamycin resistant K562/A02 cells (Fig. 1), which was consistent with drug-resistant ovarian cancer.[15] P-gp was also frequently over-expressed in leukemia.[1] A hypothesis was that STAT3 might be involved in MDR. As shown in Fig. 2 and Fig. 3, blocking of STAT3 by decoy ODN or JSI-124 increased the sensitivity of MDR cells to adriamycin and decreased the transcription of *mdr1* and expression of P-gp, so do Jak2 specific inhibitor AG490 (Figure S5). Similar results were displayed in another kind of adriamycin-resistant cell line MCF-7/ADR (Fig. S1, S2, and S3).

By using STAT3 decoy ODN or JSI-124, we verified the hypothesis that decrease the activation of STAT3 could enhance the inhibition effect of adriamycin on K562/A02 cells. The STAT3 scrambled ODN could also slightly inhibit proliferation of K562/A02 cells in our experiment (Fig. 2B), in part because it mildly inhibited the activity of STAT3 and transcription of *mdr1* (Fig. 2A, 2C). In our further research, optimizations of the STAT3 decoy/scrambled ODN and delivery system should be resulted in more profound inhibition effects. JSI-124 could dose- and time-dependent synergistically increase the adriamycin mediated proliferation inhibition in K562/A02 cells but not parental K562 cells (Fig. 3A, 3B), it was similar with the previous report in drug-resistant ovarian cancer.[15] JSI-124 could also significantly increase adriamycin-induced apoptosis (data not shown). Otherwise, STAT3C could promote the transcription of *mdr1* (Fig. 4C). ChIP and mutation assay were used to further confirm the STAT3 binding site and its functionally relevant in *mdr1* promoter. The results show that +64∼+72 region should be the binding site of STAT3 (Fig. 4B, 4D). Like other members of ABC family such as *ABCG2* and *ABCC10*, *mdr1* gene was transcribed by a TATA-less promoter, and many transcription binding sites were identified at the 5′ untranslated region (UTR), such as AP-1 and AP-2. Our research demonstrated the STAT3 binding site was also in the 5′UTR of *mdr1* gene. Further more, additional cell lines are needed to make a generalization statement related to STAT3 and *mdr1*, it was also needed to evaluate whether other STAT genes, such as STAT1 and STAT5, took part in the regulation of *mdr1*.

How was the transcription of *mdr1* regulated? Our data suggested that STAT3 could take part in regulating the transcription of *mdr1*. Regulation of *mdr1* gene transcription was complicated, and yet was not clearly known. Numerous positive and negative elements, as well as epigenetic control, were involved in the process. Several transcription factors could bind with elements of *mdr1* promoter. For example, NF-κB played an important role in regulation of *mdr1* transcription in drug resistance cancer cells.[24] The protein kinase systems, such as MAPK kinase, were associated with the regulation of *mdr1*.[25] Other signal path, such asΔNp73α, FOXO3a and hedgehog, also took part in *mdr1* transcription in gastric carcinoma and leukemia, respectively.[26], [27], [28] In a recent study, it was demonstrated that Nanog/STAT3 complex took part in the transcription of *mdr1* in breast and ovarian tumor cells.[16] Inhibition of STAT3 decreased *mdr1* mRNA in ovarian cancer cells.[29] In our present study, the ChIP and luciferase results demonstrated that STAT3 could bind to the promoter of *mdr1* and initiated its transcription, however, whether the binding was STAT3 alone or with other proteins needed to be further identified. In the recent article, Lee *et al*. reported that persistently activated STAT3 play important role in maintenance of NF-κB activity, [30] it was conjectured that STAT3 might play roles coupled with NF-κB. Whether STAT3 target genes were also associated with *mdr1* in our experiments was needed to be further identified. Recently, Bewry *et al* reported that drug resistance correlated with increased pTyrSTAT3, and resistance was associated with increased levels of the STAT3 target genes Bcl-xl, Mcl-1, and survivin.[31] In our results, we elucidated the molecular mechanism of transcription regulation of *mdr1* by STAT3.

In summary, it was demonstrated that the activation of STAT3 was higher in drug resistant K562/A02 leukemia cells, and also in MCF-7/ADR cells. And STAT3 activation inhibitors sensitized K562/A02 cells to adriamycin. STAT3 was involved in the regulation of *mdr1* transcription by binding with its promoter. Therefore, the present study provided a molecular mechanism of transcriptional regulation of *mdr1* by STAT3, and suggested that STAT3 could be potential target in treating drug resistant leukemia. In addition, our data may also be relevant for the early detection of resistance who suffering leukemia.

## Supporting Information

Figure S1 **STAT3 activation in adriamycin-resistant MCF-7/ADR cells.** **(A)** RT-PCR analysis of STAT3 and *mdr1* in MCF-7/ADR cells and MCF-7 cells.** (B)** Western blot analysis of total STAT3, phosphorylated STAT3 and P-gp levels.
(TIF)

Figure S2 **STAT3 decoy ODN increased the drug sensitivity via inhibiting the transcription of** ***mdr1*** **and increasing the accumulation of adriamycin.** (**A**) STAT3 decoy ODN increased the sensitivity of MCF-7/ADR cells to adriamycin. MCF-7/ADR cells were transfected with 100nM STAT3 decoy or scrambled ODN. After 6h, different concentrations of adriamycin were added and incubated for 48h followed by the CCK-8 assay. **(B)** STAT3 decoy ODN inhibited the transcription and expression of *mdr1*. MCF-7/ADR cells were transfected with STAT3 decoy or scrambled ODN, and mRNA and protein levels of *mdr1* were measured by real time-PCR and western blotting. (**C**) STAT3 decoy ODN increased the accumulation of adriamycin into MCF-7/ADR cells. MCF-7/ADR and MCF-7 cells were pretreated with the STAT3 decoy or scrambled ODN, and then treated with adriamycin. Fluorescence intensity of adriamycin was determined by flow cytometry.
(TIF)

Figure S3 **JSI-124 increased the sensitivity of MCF-7/ADR cells to adriamycin through down-regulation of** ***mdr1.*** **(A)** The cells were cultured with different concentrations of adriamycin combined with 0.3 µM of JSI-124 followed by the CCK-8 assay. **(B)** JSI-124 inhibited the transcription of *mdr1* and expression of P-gp. After being treated with JSI-124 as indicated concentrations for 24 h, mRNA level of *mdr1* in MCF-7/ADR cells was detected using real time-PCR. P-gp was examined by western blotting after being treated for 36h. (**C**) JSI-124 increased the accumulation of adriamycin into MCF-7/ADR cells. Cells were pretreated with JSI-124 for 24h, and then treated with adriamycin for 90min. Fluorescence intensity were determined by flow cytometry.
(TIF)

Figure S4 **JSI-124 increased the sensitivity of K562/A02 cells to daunorubicin.** K562/A02 cells were treated with different concentrations of daunorubicin combined with 1 µM of JSI-124 for 48h followed by the CCK-8 assay.
(TIF)

Figure S5 **Jak2 specific inhibitor AG490 enhanced the drug sensitivity of K562/A02 cells via down-regulating P-gp. (A)** The K562/A02 cells were treated with different concentrations of adriamycin in the presence of 100 µM/50 µM of AG490 for 48h followed by the CCK-8 assay. An average of at least three triplicates from three separate experiments was calculated. The inhibition ratio was calculated by following formula: inhibition (%)  =  (1-experimental OD/control OD) ×100%. **(B)** mRNA level of MDR1 was detected using real time-PCR method. Relative expressions were calculated. **(C)** After being treated with AG490 at indicated concentrations for 36h, the whole-cell extracts were obtained. P-gp, pSTAT3 (Y705) and STAT3 were then examined by western blotting.
(TIF)

Table S1 **The primer sequences for real time-PCR.**
(DOC)
