# Supplementary figures and images for: Deactivation of Signal Transducer and Activator of Transcription 3 Reverses Chemotherapeutics Resistance of Leukemia Cells via Down-Regulating P-gp
Source: PLoS One. 2011 Jun 6;6(6):e20965. doi: 10.1371/journal.pone.0020965 (PMC3108986; doi:10.1371/journal.pone.0020965)

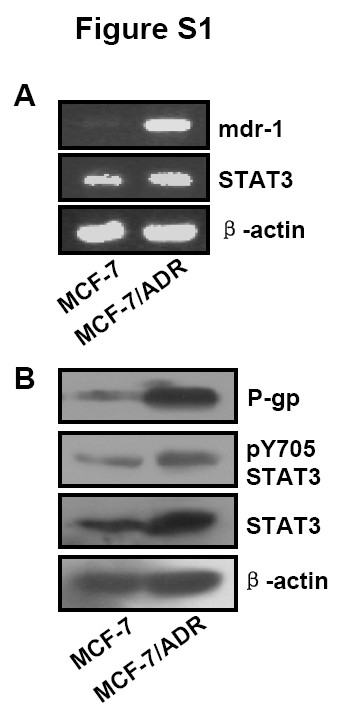

Supplement: Figure S1 — STAT3 activation in adriamycin-resistant MCF-7/ADR cells. (A) RT-PCR analysis of STAT3 and mdr1 in MCF-7/ADR cells and MCF-7 cells. (B) Western blot analysis of total STAT3, phosphorylated STAT3 and P-gp levels. (TIF) [file pone.0020965.s001.tif]

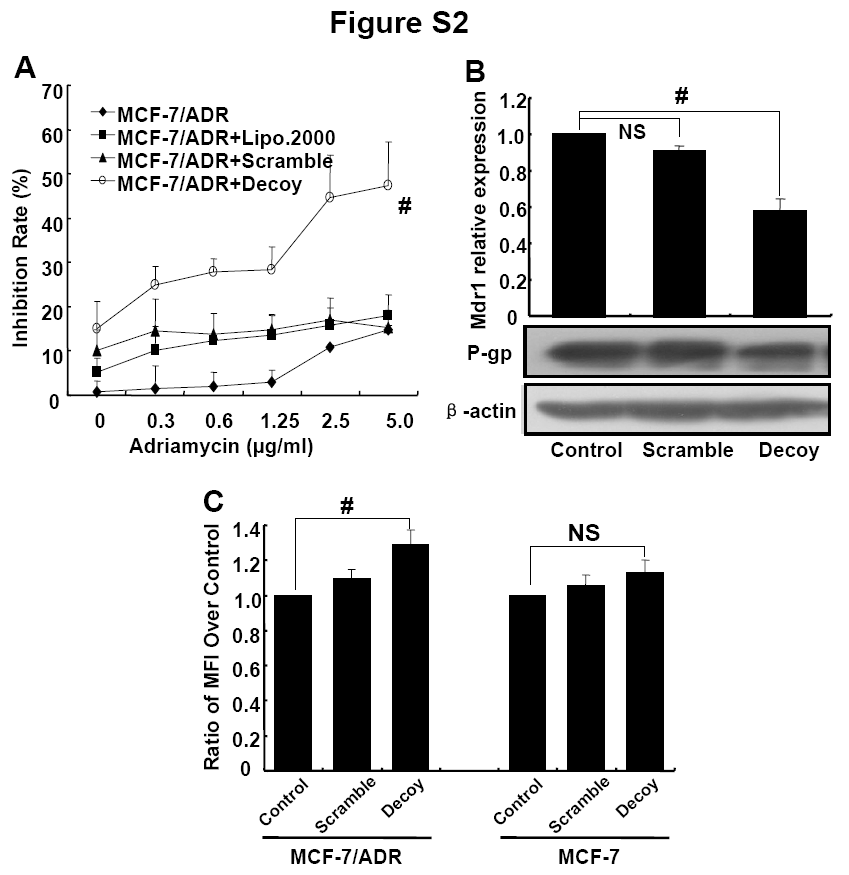

Supplement: Figure S2 — STAT3 decoy ODN increased the drug sensitivity via inhibiting the transcription of mdr1 and increasing the accumulation of adriamycin. (A) STAT3 decoy ODN increased the sensitivity of MCF-7/ADR cells to adriamycin. MCF-7/ADR cells were transfected with 100nM STAT3 decoy or scrambled ODN. After 6h, different concentrations of adriamycin were added and incubated for 48h followed by the CCK-8 assay. (B) STAT3 decoy ODN inhibited the transcription and expression of mdr1. MCF-7/ADR cells were transfected with STAT3 decoy or scrambled ODN, and mRNA and protein levels of mdr1 were measured by real time-PCR and western blotting. (C) STAT3 decoy ODN increased the accumulation of adriamycin into MCF-7/ADR cells. MCF-7/ADR and MCF-7 cells were pretreated with the STAT3 decoy or scrambled ODN, and then treated with adriamycin. Fluorescence intensity of adriamycin was determined by flow cytometry. (TIF) [file pone.0020965.s002.tif]

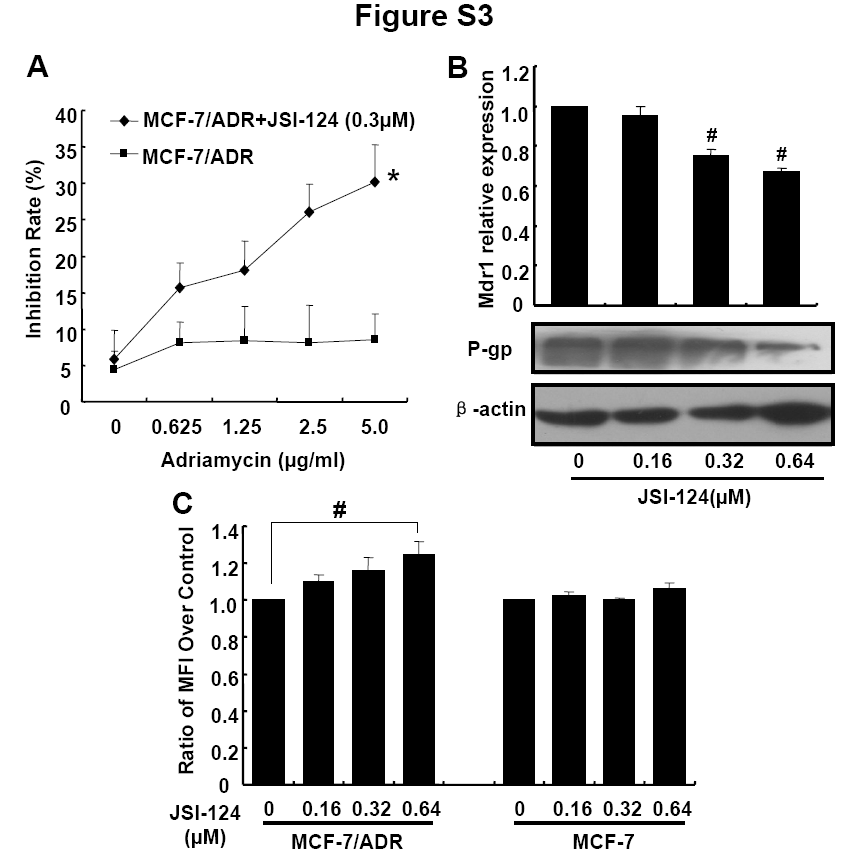

Supplement: Figure S3 — JSI-124 increased the sensitivity of MCF-7/ADR cells to adriamycin through down-regulation of mdr1. (A) The cells were cultured with different concentrations of adriamycin combined with 0.3 µM of JSI-124 followed by the CCK-8 assay. (B) JSI-124 inhibited the transcription of mdr1 and expression of P-gp. After being treated with JSI-124 as indicated concentrations for 24 h, mRNA level of mdr1 in MCF-7/ADR cells was detected using real time-PCR. P-gp was examined by western blotting after being treated for 36h. (C) JSI-124 increased the accumulation of adriamycin into MCF-7/ADR cells. Cells were pretreated with JSI-124 for 24h, and then treated with adriamycin for 90min. Fluorescence intensity were determined by flow cytometry. (TIF) [file pone.0020965.s003.tif]

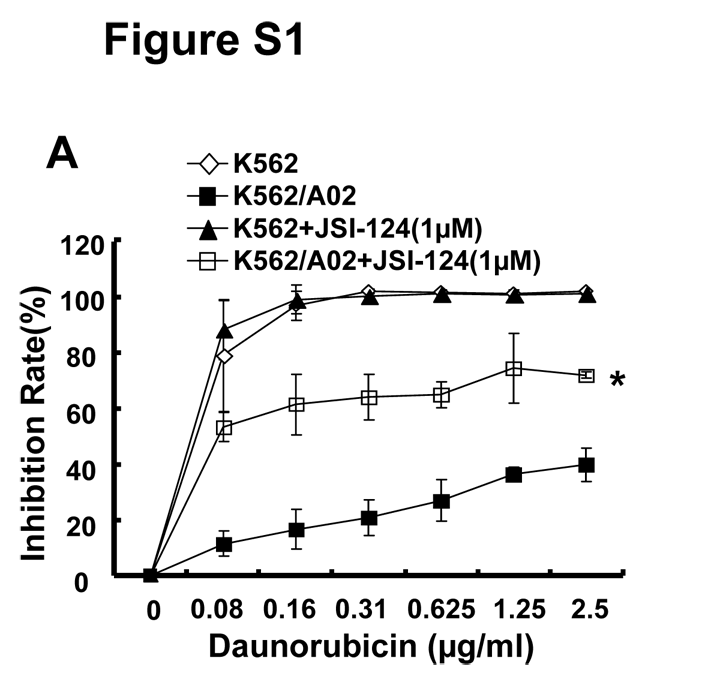

Supplement: Figure S4 — JSI-124 increased the sensitivity of K562/A02 cells to daunorubicin. K562/A02 cells were treated with different concentrations of daunorubicin combined with 1 µM of JSI-124 for 48h followed by the CCK-8 assay. (TIF) [file pone.0020965.s004.tif]

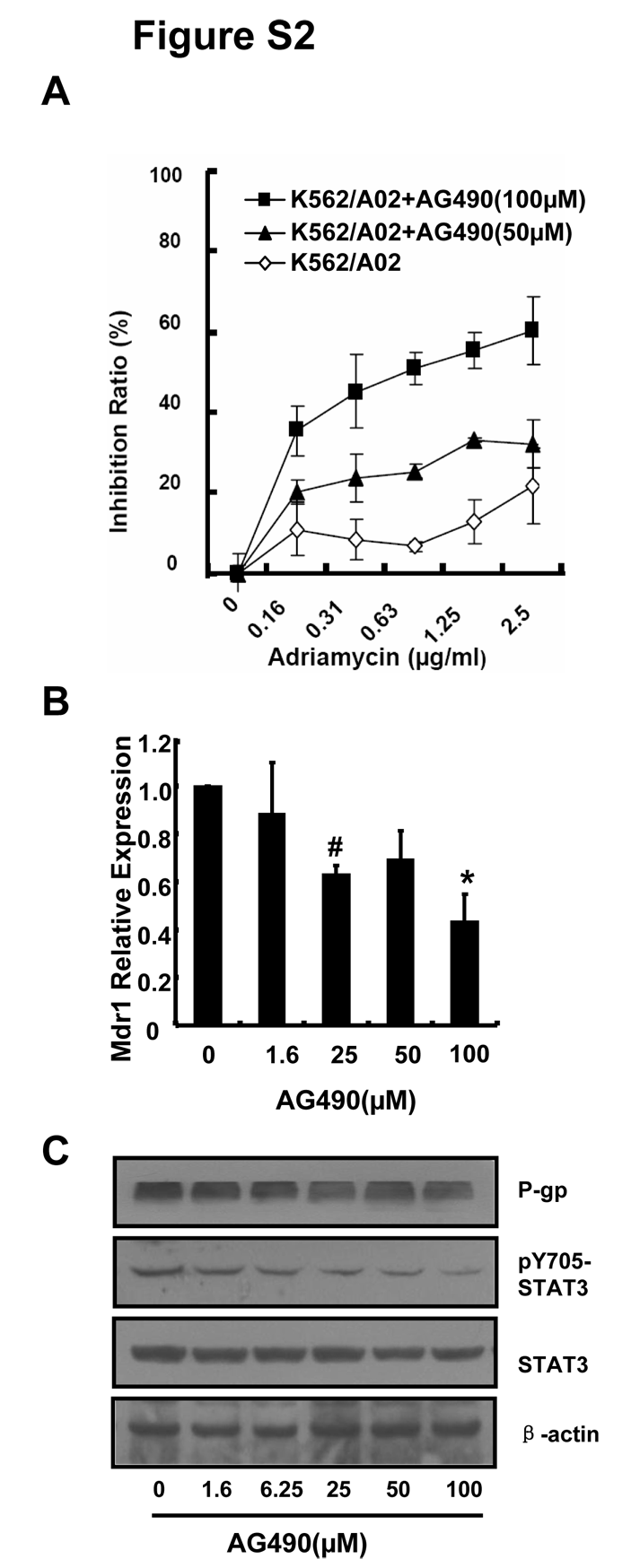

Supplement: Figure S5 — Jak2 specific inhibitor AG490 enhanced the drug sensitivity of K562/A02 cells via down-regulating P-gp. (A) The K562/A02 cells were treated with different concentrations of adriamycin in the presence of 100 µM/50 µM of AG490 for 48h followed by the CCK-8 assay. An average of at least three triplicates from three separate experiments was calculated. The inhibition ratio was calculated by following formula: inhibition (%) = (1-experimental OD/control OD) ×100%. (B) mRNA level of MDR1 was detected using real time-PCR method. Relative expressions were calculated. (C) After being treated with AG490 at indicated concentrations for 36h, the whole-cell extracts were obtained. P-gp, pSTAT3 (Y705) and STAT3 were then examined by western blotting. (TIF) [file pone.0020965.s005.tif]
