# Supplementary material for: Deactivation of Signal Transducer and Activator of Transcription 3 Reverses Chemotherapeutics Resistance of Leukemia Cells via Down-Regulating P-gp
Source: PLoS One. 2011 Jun 6;6(6):e20965. doi: 10.1371/journal.pone.0020965 (PMC3108986; doi:10.1371/journal.pone.0020965)
Supplement: Table S1 — The primer sequences for real time-PCR. (DOC) [file pone.0020965.s006.doc]

**SUPPORTING TABLES**

**Table S**1. The primer sequences for real time-PCR.

| Gene |  | Primer sequence |
| --- | --- | --- |
| β-actin | sense | 5’-CACTGTGTTGGCGTACAGGT-3’ |
|  | antisense | 5’-TCATCACCATTGGCAATGAG-3’ |
| Mdr1 | sense | 5’-TGACATTTATTCAAAGTTAAAAGCA-3’ |
|  | antisense | 5’-TAGACACTTTATGCAAACATTTCAA-3’ |
